# Design and Implementation of a SiC-Based Multifunctional Back-to-Back Three-Phase Inverter for Advanced Microgrid Operation

**DOI:** 10.3390/mi14010134

**Published:** 2023-01-03

**Authors:** Chao-Tsung Ma, Zhi-Yuan Zheng

**Affiliations:** Applied Power Electronics Systems Research Group, Department of EE, CEECS, National United University, Miaoli City 36063, Taiwan

**Keywords:** wide bandgap (WBG) semiconductor, microgrid (MG), distributed generation (DG), renewable energy (RE), static switch (SS), back-to-back (BTB) inverter

## Abstract

Because of the worldwide trend of microgrid (MG) and renewable energy (RE)-based distributed power generation (DG), advanced power flow control schemes with wide bandgap (WBG) semiconductor technologies to ensure high-level performance of grid-connected MGs is one of the crucial research topics. In grid-connected MGs, a static switch (SS) is commonly used at the point of common coupling (PCC) of two systems. In this paper, the role of SS is replaced by a SiC-based three-phase back-to-back (BTB) inverter system for seamless switching between grid-connected and standalone modes through advanced power flow control schemes. According to scenarios of different grid/load conditions and available DG capacities in an MG, various advanced control functions can be developed for both MG operating modes: bidirectional control of active and reactive power flows, seamless switching between operating modes, improvement of grid power quality (PQ), and voltage stabilization. In this paper, mathematical models of the BTB inverter in a synchronous reference frame (SRF) is first derived, and the required controllers are then designed. For functional testing, two typical cases are simulated and analyzed in a MATLAB/Simulink environment and then verified through 1kVA small-scale hardware implementation with Texas Instruments (TI) digital signal processor (DSP) TMS320LF2812 as the control core. Results show satisfactory performances of power flow control and PQ improvement of MG.

## 1. Introduction

Because of the worldwide trend of carbon reduction and green energy, RE-based DG and MGs have been extensively researched in order to better support modern industries with reliable power systems. RE offers clean and inexhaustible power sources such as solar photovoltaic (PV) and wind energies, and MGs offer lower construction and power distribution costs and better PQ management, operation flexibility, and maintenance accessibility. The integration of MGs and DG is an inevitable trend for future power generation and optimization for both industries and communities [1,2,3,4]. In the initial stages of MG development, common loads were mostly AC loads, so related research was focused on AC MGs, where power rectifiers were required when supplying DC loads. Later, in order to respond to the increasing need to supply DC loads, researchers also started to pay more attention to DC MGs, where power inverters were required when supplying AC loads. Consequently, these two approaches both increased cost and decreased convenience. As a result, hybrid AC/DC MGs (HMGs) were developed to simultaneously supply DC and AC loads without extra power converters and allow better integration of various DG systems [5,6,7,8,9,10,11,12]. Since the HMG is a future trend, a lot of discussion and many studies have been published in recent years. In terms of the key technologies for HMGs, F. Nejabatkhah et al. [13] provided an overview on PQ control of real-world smart HMGs, where the decentralized architecture was found advantageous in the aspects of DG, energy storage, and load management. In [14], the application of fuzzy logic control to HMGs was reviewed. It was pointed out that the strong control capability of fuzzy logic was extremely useful in such a multi-agent system compared with conventional control methods, and that fuzzy-based intelligent clustered MG operation would be a future trend. A two-level distributed control architecture was proposed for the economic operation of global HMGs in [15]. Incremental cost droop control was employed in the first level to minimize the cost, and a distributed control canonical form was proposed in the second level to solve the issue of AC frequency and DC link voltage deviation due to the droop control in the first level. A. Agrawal and R. Gupta [16] proposed a flexible distributed power sharing coordination control based on multiple stacked interfacing converter and interlinking converter for HMGs with various energy sources. The proposed controller offered the advantage of minimal parameter tuning requirements and the integration of a centralized battery bank. In [17], a two-layer control was proposed for stable frequency and optimal economic operation of HMGs. The lower layer dealt with economic power dispatch, while the upper layer performed power regulation between DC and AC sections through interlinking converters.

In practice, the MG may operate in standalone mode and grid-connected mode. In standalone mode, all the loads are supplied locally using DG and stored energy. In grid-connected mode, the grid can assist the MG in overcoming PQ issues and supply electricity when DG and stored energy are insufficient to supply local loads; an MG can also feed excessive electricity generation to the grid. The most important job of an MG is to make sure critical loads are not affected by grid faults such as voltage sags, which can easily and almost instantly harm sensitive loads. As a result, an SS based on power electronic switching devices is necessary for the coupling of MGs and grids at the PCC. After the fault is eliminated, re-synchronization of voltage amplitude, frequency, and phase is necessary for reconnection, which can be optimally achieved using a power electronic-based SS. The use of SS also allows the grid to consider each MG a single dispatchable unit; each unit has the ability to supply excessive electricity and draw power from the grid when necessary [18,19]. E. Pashajavid et al. [20] proposed a self-healing strategy for improved overloading resilience of two standalone MGs connected with an SS, where the two MGs supported each other. Centralized and decentralized operations were developed for when data communication was available and not, respectively. In [21], a controller for SS was designed based on Park’s transformation, multiresolution analysis, and Takagi–Sugeno–Kang fuzzy rules. Faults were effectively detected and classified without the need for a complex communication. The connection between a solar PV generation system and a power grid using SS was studied in [22]. The operating point can be effectively controlled by the SS according to given temperature and irradiance. Further current harmonic suppression was still required. G.H. Gwon et al. [23] proposed a low-cost load transfer SS to solve the problem of spontaneous voltage imbalance due to neutral current in a bidirectional LV DC distribution system. The neutral current at each load point was taken into consideration. System efficiency was successfully improved.

**Figure 1:**
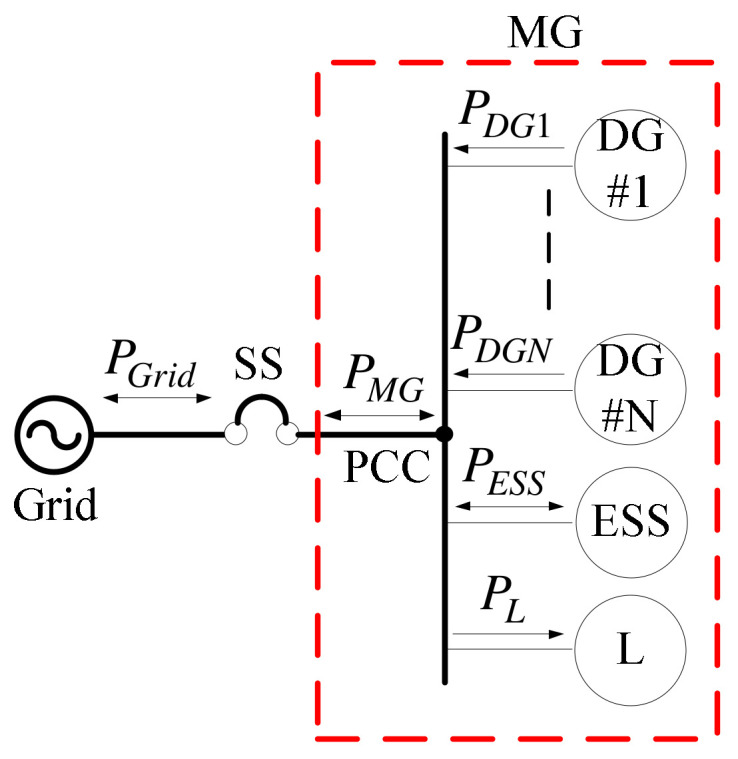
Architecture of a typical grid-connected MG.

Currently, the designs of practical MGs mostly target at grid-connected applications. At the PCC, which is the responsibility division point of two systems, an SS is usually used to control the operation modes and protect both systems. Figure 1 shows the architecture of a typical grid-connected MG, where *P_Grid_* denotes grid power, *P_MG_* denotes MG power, *P_DGn_* denotes power of each DG system, *P_ESS_* denotes ESS power, and *P_L_* denotes load power. As the adoption of MGs increases year by year, power flow control and power management of MGs are also becoming particularly important. In addition, when DG systems are integrated into an existing grid, the previously conventional (radial) distribution system will be transformed into a multi-power supply distribution system, and the magnitude and direction of power flows within will change substantially. In particular, DG systems are mostly interfaced using power electronics; without proper circuitry and control strategy, a DG system will be unstable and prone to failure. In recent years, with the continuous advancement of related technologies, many high-tech industries are demanding increasingly better PQ. Poor PQ can easily lead to faults, tripping, shortened life, and even damage. However, since power electronic equipment is mostly controlled with high-frequency pulse width modulation (PWM), power contamination caused by harmonics is quite serious. Therefore, constructing a stable and high-quality power system with advanced power electronics technologies is an extremely important task for modern distribution systems.

**Figure 2:**
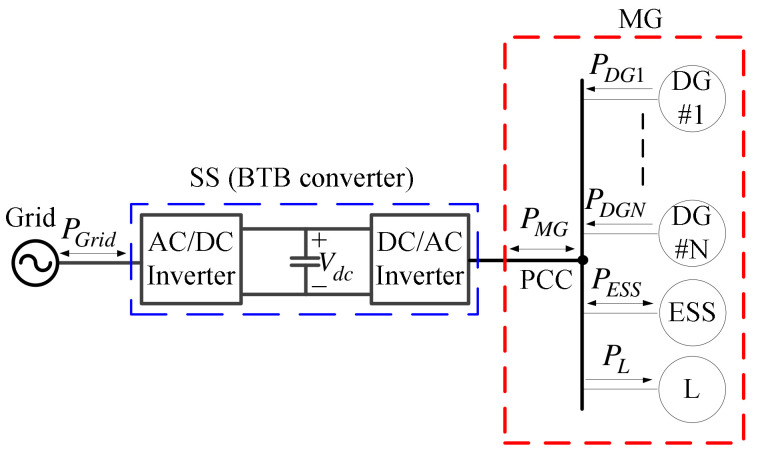
A grid-connected MG with a BTB inverter-based SS.

Based on the above, this paper proposes a SiC-based back-to-back (BTB) three-phase inverter to replace the SS, as shown in Figure 2, for the advanced operation and control of grid-connected MGs. The proposed BTB inverter is capable of power flow control and management, which can be used to isolate the MG from the grid when fault occurs, so that low-quality power is kept from disturbing other systems. This topology has the advantages of high power factor (PF) and high efficiency, reduced system volume, and capability of bidirectional power flow, while its obvious disadvantage is electromagnetic interference (EMI) due to the high switching frequency used, which may lead to the requirement of proper EMI filters. Three-phase inverters are commonly used in applications such as AC sources, uninterruptible power supplies (UPS), motor drives, MGs, wind turbine generators (WTG), and electric transportation [24,25,26]. I. Abdelsalam et al. [27] proposed a new dual current source BTB converter for medium-voltage/multi-MW WTGs with a wide wind speed range and good dynamics. Pulsating torque was eliminated using diode rectifiers combined with a phase-shift transformer. The design reduced circuitry and control complexity, where active and reactive powers were controlled independently, and loss, achieving zero loss for the grid inverter. In [28], a 9 kW 150 kHz silicon carbide (SiC)-based sinusoidal PWM (SPWM) three-phase four-wire BTB inverter was proposed. Zero-voltage switching (ZVS) was achieved under both balanced and unbalanced load conditions using only one auxiliary circuit, which only operated once for each turn-on process, thus reducing extra circuitry losses (over 3%). Furthermore, the voltage stress was substantially reduced. A “finite-control-set model predictive control” was proposed in [24] for a three-phase four-leg eight-switch BTB inverter-fed induction motor drive to solve the issue of voltage deviation between the two capacitors. Fast tracking of torque and flux commands, bidirectional power flow, and power PF control were achieved. L. Shen et al. [25,29] proposed an active DC-link capacitor harmonic current self-cancellation method based on harmonic phase feedback control and by controlling PWM phase angles for a two-level three-phase BTB converter, enhancing the reliability of BTB converters. In [26], a PWM-based common monde (CM) voltage cancellation strategy for two-level three-phase BTB inverters was proposed by synchronizing the commutation of both converters. This method was tested on a 15 kW motor drive. More than 15 dB reduction was achieved up to a few hundred kHz switching frequency. A circulating current reduction method was proposed in [30] using SPWM-based division-summation digital control for a 10 kVA three-phase BTB transformer-less inverter in online uninterruptable power supply (UPS) application. DC link voltage variation, grid voltage distortion, and inductance variation were all taken into consideration. The three-phase inductor currents were controlled independently. Significant reduction of the weight, size, and cost were achieved.

In order to reduce high-frequency harmonic contamination, the SiC-based three-phase back-to-back (BTB) inverter designed in this paper uses a third-order LCL filter. Mathematical modeling of the BTB inverter and its PI-based controllers in SRF is first explained in the next section. In the third section, the quantification design of necessary controllers is carried out. In the fourth section, MATLAB/Simulink is first used to simulate the proposed system, and then TI DSP TMS320LF2812 is used as the control core for hardware tests and verification. Two typical cases for both simulation and implementation tests are used to demonstrate the capabilities of the proposed BTB inverter. Results show satisfactory performance of the proposed inverter in terms of power flow control and PQ improvement.

## 2. Mathematical Modeling of Proposed BTB Inverter and Controllers

**Figure 3:**
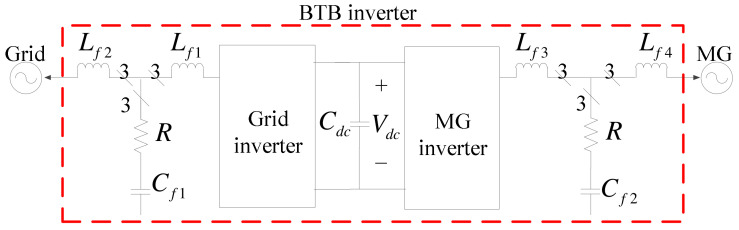
The circuit configuration of the proposed BTB inverter-based SS.

The BTB inverter-based hardware system proposed in this paper consists of two SiC-based three-phase voltage source inverters (VSIs) with the same configuration for both grid and MG sides, as shown in Figure 3. The LCL filters are used to reduce harmonic currents caused by high-frequency PWM. In addition to ensuring good PQ of the MG, the proposed inverter system is also capable of power management and can adjust active and reactive powers according to real-time requirements.

The main functions of the grid inverter are to stabilize DC link voltage and, if necessary, perform reactive power compensation. Other important functions include suppressing voltage flicker, preventing voltage sags, and improving system PF, in order to reduce power loss and enhance system stability. The MG inverter can compensate for insufficient power generation in the MG according to load and generation conditions. On the other hand, when the DG in the MG outputs excessive active power, the MG inverter can also perform energy storage using the ESS in the MG, or the excessive power can also be fed to the grid through both inverters. In terms of reactive power regulation, in addition to supplying load power and maintaining unity PF (UPF), the reactive power compensation function can be performed to maintain voltage stability of the MG and improve system efficiency. In this paper, dq-axis current decoupling control in SRF is adopted, and conventional P and PI controllers are used. Effective reduction of the mutual influence between the d and q axes is achieved.

### 2.1. Grid Inverter

**Figure 4:**
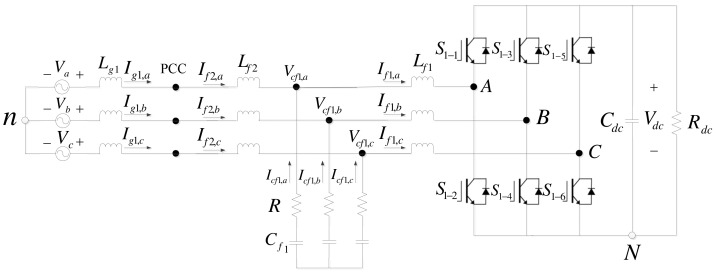
Configuration of the grid inverter.

Figure 4 shows the architecture of the grid inverter, where *L_g1_* denotes grid inductances, *L_f1_* and *L_f2_* denote filter inductors, *C_f1_* denotes filter capacitors, *R* denotes damping resistors, *S_1-1_* through *S_1-6_* denote semiconductor switches, *C_dc_* denotes DC link capacitor, *R_dc_* denotes equivalent DC load, *n* denotes grid ground, *V_a_*, *V_b_*, and *V_c_* denote grid three-phase voltages, *I_g1a_*, *I_g1b_*, and *I_g1c_* denote grid three-phase line currents, *I_f1,a_*, *I_f1,b_*, and *I_f1,c_* denote three-phase line currents through *L_f1_*, *I_f2,a_*, *I_f2,b_*, and *I_f2,c_* denote three-phase line currents through *L_f2_*, *V_cf,a_*, *V_cf,b_*, and *V_cf,c_* denote three-phase voltages across *C_f1_*, *I_cf,a_*, *I_cf,b_*, and *I_cf,c_* denote three-phase line currents through *C_f1_*, and *V_dc_* denotes DC link voltage.

**Figure 5:**
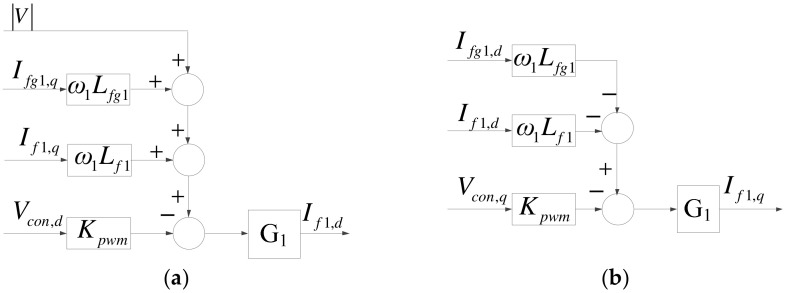
Mathematical models of grid inverter currents: (**a**) d axis; (**b**) q axis.

Assuming the condition of three-phase balance, we can use Kirchhoff’s voltage law and Park’s transformation to obtain (1) and (2), which yield Figure 5.

(1) $$I_{f1,d}=G_{1}(-K_{pwm}V_{con,d}+\omega_{1}L_{f1}I_{f1,q}+\omega_{1}L_{fg1}I_{fg1,q}+\left|V\right|)$$

(2) $$I_{f1,q}=G_{1}(-K_{pwm}V_{con,q}-\omega_{1}L_{f1}I_{f1,d}-\omega_{1}L_{fg1}I_{fg1,d}),$$

where

(3) $$G_{1}=\frac{S^{2}C_{f1}L_{fg1}+SC_{f1}R+1}{S^{3}C_{f1}L_{f1}L_{fg1}+S^{2}C_{f1}R(L_{f1}+L_{fg1})+S(L_{f1}+L_{fg1})},$$

(4) $$L_{fg1}=L_{f2}+L_{g1},$$

(5) $$I_{fg1,q}=\frac{SC_{f1}R+1}{S^{2}C_{f1}L_{fg1}+SC_{f1}R+1}I_{f1,q},$$

(6) $$I_{fg1,d}=\frac{SC_{f1}R+1}{S^{2}C_{f1}L_{fg1}+SC_{f1}R+1}I_{f1,d},$$

*K_pwm_* = *V_dc_*/2*V_tri_* (*V_tri_* denotes carrier amplitude), and *V_con,d_* and *V_con,q_* denote dq-axis voltage control signals.

**Figure 6:**
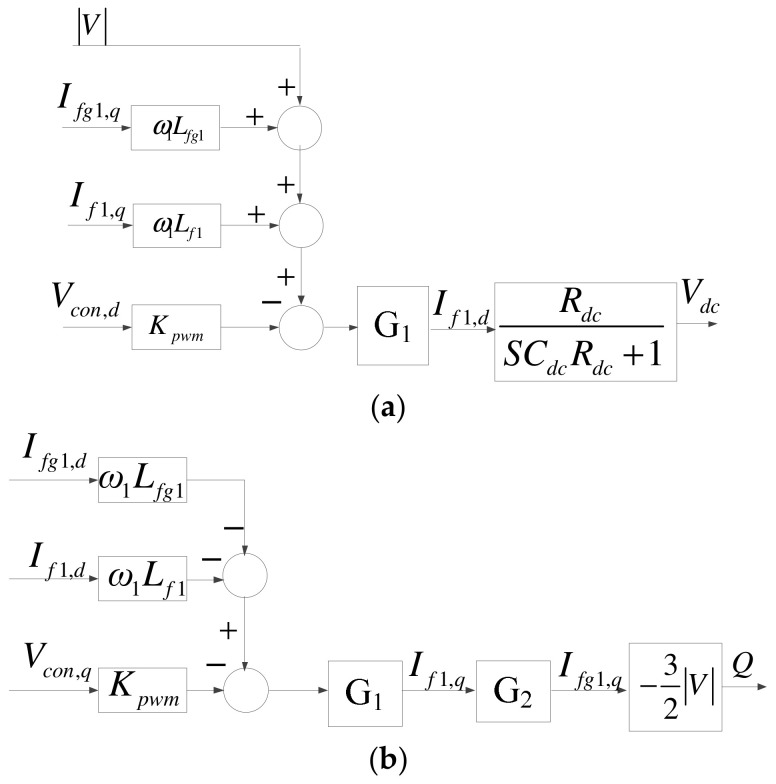
Q-axis mathematical model of the grid inverter: (**a**) d axis; (**b**) q axis.

Since there is only active current in the DC interface, DC link voltage can be controlled solely through *I_f1,d_*. Therefore, the transfer function of DC link voltage is as shown in (7), and thus the complete d-axis mathematical model of the grid inverter is as shown in Figure 6a. The reactive power of the grid inverter can be controlled through *I_fg1,q_*. As a result, the complete q-axis mathematical model of the grid inverter can be obtained as shown in Figure 6b, where G_2_ is shown in (8).

(7) $$V_{dc}=\frac{R_{dc}}{SC_{dc}R_{dc}+1}I_{f1,d}.$$

(8) $$G_{2}=\frac{SC_{f1}R+1}{S^{2}C_{f1}L_{fg1}+SC_{f1}R+1}.$$

### 2.2. Grid Inverter Controllers

**Figure 7:**
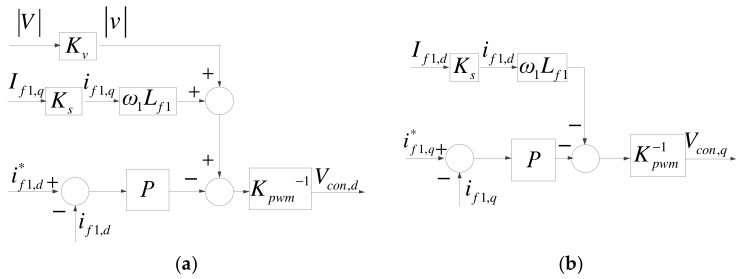
Grid inverter inner-loop current controllers: (**a**) d axis; (**b**) q axis.

The control architecture of grid inverter consists of inner-loop current controllers and outer-loop DC link voltage/reactive power controllers, and conventional P and PI controllers are employed. First, the current controllers are derived according to Figure 4. In the case of three-phase balance, current control equations can be obtained through Park’s transformation, as shown in (9) and (10), and their mathematical models are shown in Figure 7.

(9) $$V_{con,d}=-K_{pwm}{\;}^{-1}[L_{f1}\frac{di_{f1,d}}{dt}-\omega_{1}L_{f1}i_{f1,q}-\left|v\right|];$$

(10) $$V_{con,q}=-K_{pwm}{\;}^{-1}[L_{f1}\frac{di_{f1,q}}{dt}+\omega_{1}L_{f1}i_{f1,d}].$$

**Figure 8:**
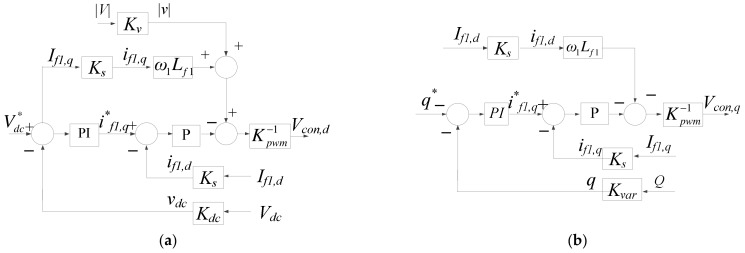
Grid inverter outer-loop controllers: (**a**) DC link voltage controller (d axis); (**b**) reactive power controller (q-axis).

**Figure 9:**
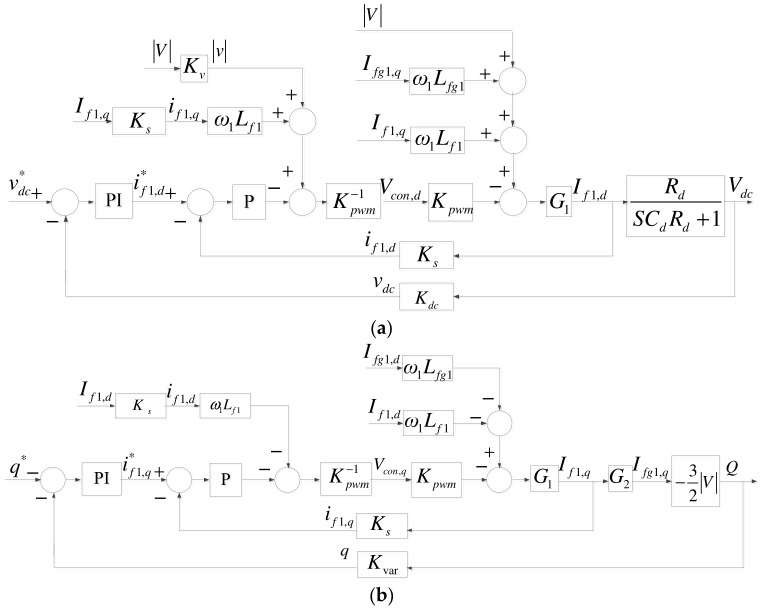
Complete grid inverter control loop: (**a**) d axis; and (**b**) q axis.

DC link voltage/reactive power controllers both adopt P and PI controllers, with DC link voltage sampling scale *K_dc_*, AC voltage sampling scale *K_v_*, AC current sampling scale *K_s_*, and reactive power sampling scale *K_var_*. According to the above derivation, mathematical models of the DC link voltage/reactive power controllers can be obtained, as shown in Figure 8. As a result, complete mathematical models of grid inverter under the two axes are shown in Figure 9.

### 2.3. MG Inverter

**Figure 10:**
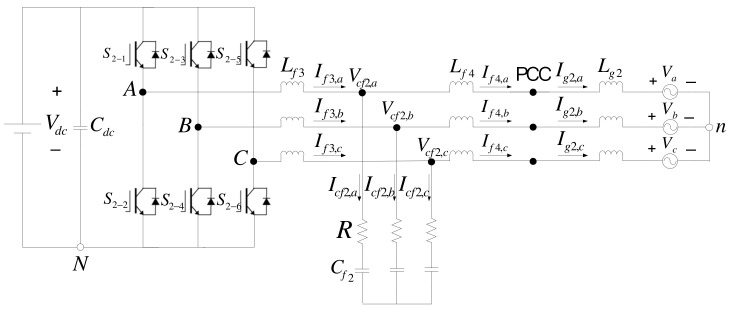
Configuration of the MG inverter.

**Figure 11:**
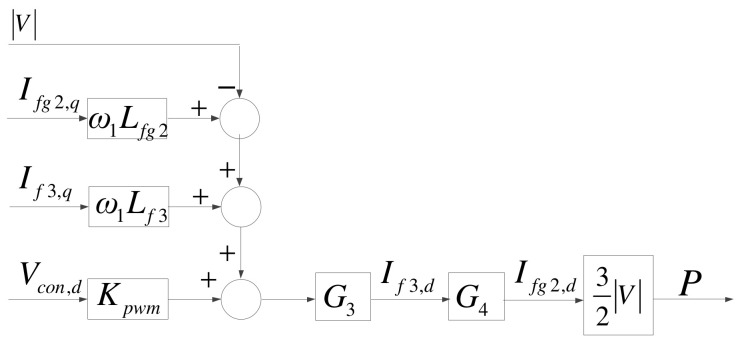
D-axis mathematical model of the MG inverter.

The architecture of the MG inverter is identical to that of the grid inverter, as shown in Figure 10. However, the outputs of the MG inverter include both active and reactive powers; therefore, its d-axis mathematical model is used to control active power and is slightly different from that of the grid inverter, as shown in Figure 11, where G_3_ is identical to G_1_, and G_4_ is identical to G_2_.

### 2.4. MG Inverter Controllers

**Figure 12:**
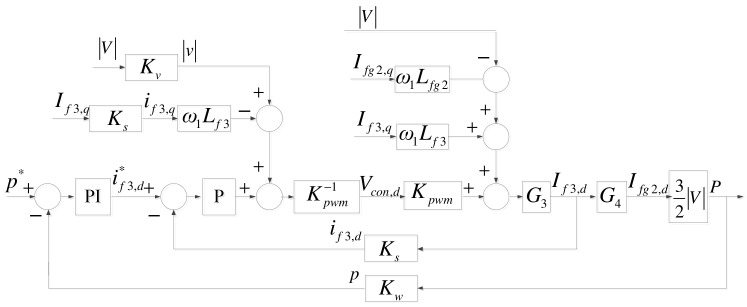
Complete MG inverter d-axis control loop.

MG inverter controllers are also similar to grid inverter controllers. The only modification required is replacing the grid inverter DC link voltage controller (d-axis) with an active power controller, as shown in Figure 12.

## 3. Quantification Design of Proposed Controllers

The inverter system specifications designed in this paper are as listed in Table 1. The design process in this section will be carried out according to these specifications.

### 3.1. Grid Inverter Controllers

**Figure 13:**
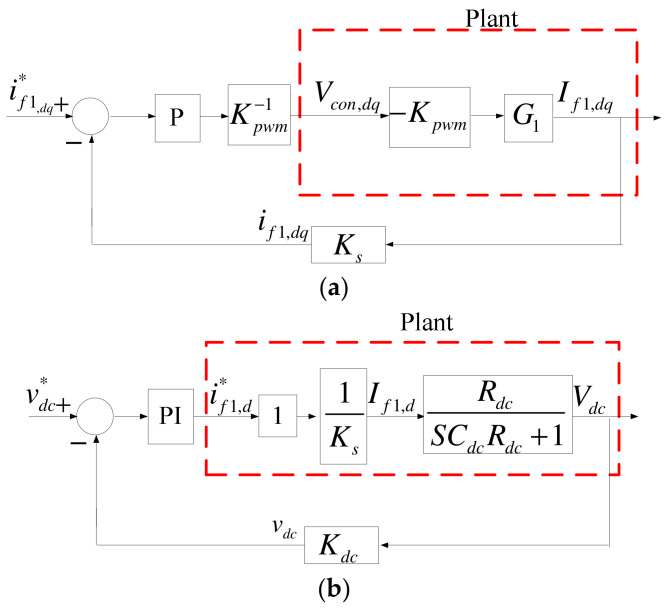

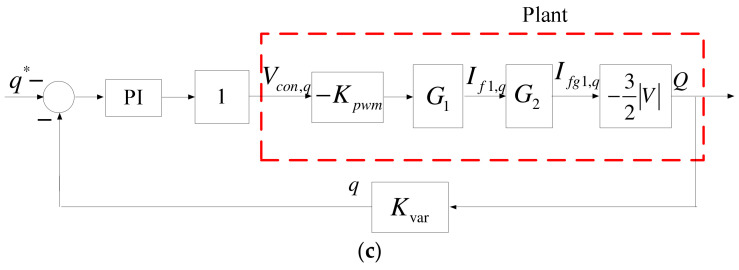
(**a**) Inner-loop current controller of grid inverter; (**b**) simplified outer-loop DC link voltage controller of grid inverter; and (**c**) simplified outer-loop DC link voltage controller of grid inverter.

Letting *−K_pwm_V_con,dq_* = *e_dq_* and using (11) we can obtain the inner current control loop of grid inverter as shown in Figure 13a. The angular frequency is designed at 3.37 × 10^3^ rad/s, and thus we get the amplitude of −38.1 dB and gain of 80, which is then multiplied by $K_{pwm}^{-1}$ (0.025) to obtain the proportional gain of 2. In terms of DC link voltage controller, since the bandwidth of current controller is much wider than that of the DC link voltage controller, it can be considered as 1 during the design of outer-loop DC link voltage controller. Therefore, Figure 13a is simplified to obtain the mathematical model of grid inverter DC link voltage controller shown in Figure 13b. The plant transfer function can be expressed as (12). Designing the angular frequency at 37 rad/s, we get an amplitude of −9.5 dB and proportional gain of 3, and thus *K_I_* can be calculated to be 1110. For a reactive power controller, Figure 9b can be simplified into Figure 13c. Letting *−K_pwm_V_con,q_* = *e_q_*, we can obtain control plant transfer Function (13). The design point is chosen at 190 rad/s angular frequency, where the amplitude is −4 dB, and *K_p_* = 1.6. *K_I_* is then calculated to be 3040.

(11) $$\frac{i_{f1,dq}}{e_{dq}}=K_{s}\times \frac{S^{2}C_{f1}L_{fg1}+SC_{f1}R+1}{S^{3}C_{f1}L_{f1}L_{fg1}+S^{2}C_{f1}R(L_{f1}+L_{fg1})+S(L_{f1}+L_{fg1})}.$$

(12) $$\frac{v_{dc}}{i_{f1,d}^{*}}=\frac{K_{dc}}{K_{s}}\times \frac{R_{dc}}{SC_{dc}R_{dc}+1}.$$

(13) $$\frac{-q}{e_{q}}=\frac{3}{2}\left|V\right|K_{\mathrm{var}}\times \frac{SC_{f1}R+1}{S^{3}C_{f1}L_{f1}L_{fg1}+S^{2}C_{f1}R(L_{f1}+L_{fg1})+S(L_{f1}+L_{fg1})}.$$

To sum up the design, the results of controller parameters are listed in Table 2.

### 3.2. MG Inverter Controllers

The design of MG inverter controllers is the same as in the previous subsection. The specifications are also similar to those of grid inverter controllers. The difference is that active power sampling factor *K_w_* is added and equals 0.002. The design results are listed in Table 3.

## 4. Simulation and Implementation

**Figure 14:**
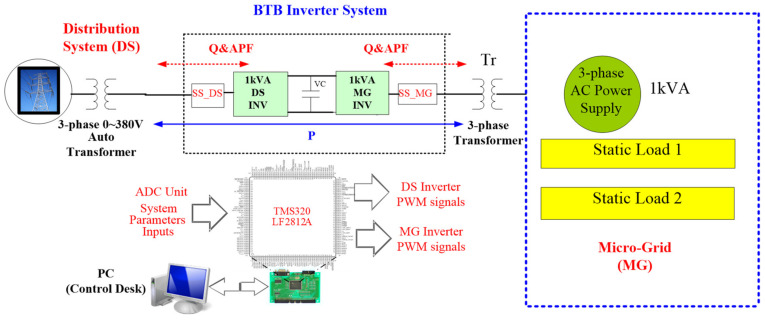
Configuration of the proposed experimental system.

For system verification objectives, simulation and implementation are conducted based on the configuration shown in Figure 14. The system is simulated in a MATLAB/Simulink environment, and then software–hardware integration implementation is performed with TI DSP TMS320LF2812 as control core. TMS320LF2812 is the industry’s first 32 bit control chip, with flash memory and up to 150 million of instructions per second (MIPS) calculating bandwidth; it belongs to C28x series, which are specifically designed for industry and automation control with complex control algorithms. As can be seen in Figure 14, with the proposed configuration the BTB inverter is capable of performing many functions, including bidirectional active/reactive power flow control, seamless operating mode switching, grid PQ improvement and voltage stabilization, MG PQ improvement and reactive power compensation. However, in order to avoid making this paper too long, only two cases are elaborated here for demonstration purposes. The first case focuses on active/reactive power control of MG inverter, and the second case focuses on PQ improvement control.

### 4.1. Simulation Results

#### 4.1.1. Case 1

**Figure 15:**
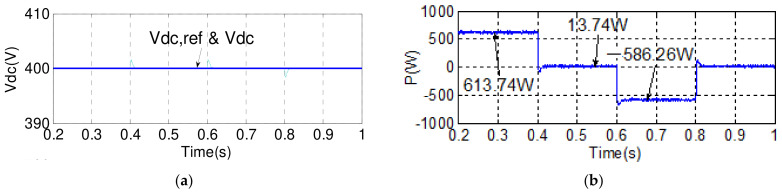

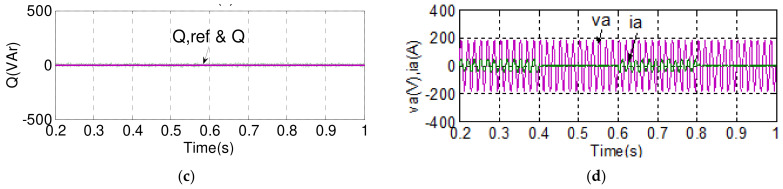
Simulated grid inverter waveforms in case 1: (**a**) DC link voltage and reference; (**b**) active power; (**c**) reactive power and reference; (**d**) phase-a voltage and current waveforms.

**Figure 16:**
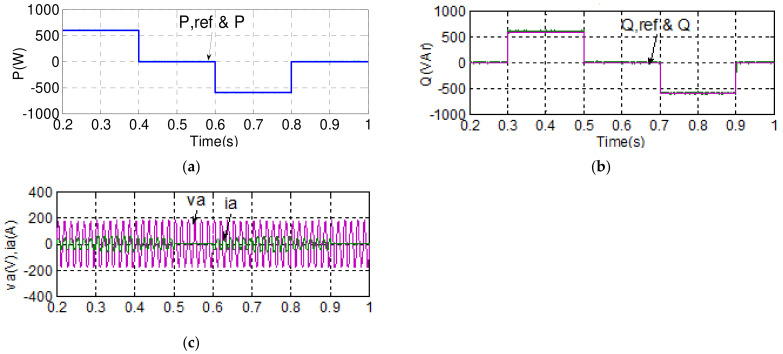
Simulated MG inverter waveforms in case 1: (**a**) active power and reference; (**b**) reactive power and reference; (**c**) phase-a voltage and current waveforms.

To demonstrate the real-time power flow control function using the proposed BTB inverter between the grid and MG, the simulation case 1 explores a set of active/reactive power flow tracking control results of both grid and MG inverters under normal voltage conditions. The simulation scenario is described as follows: grid inverter maintains the DC link voltage at 400 V and reactive power at 0 VAR; active power command for the MG inverter is set at 600 W during 0.2–0.4 s, 0 W during 0.4–0.6 s, −600 W during 0.6–0.8 s, and 0 W during 0.8–1 s, while reactive power command for the MG inverter is set at 0 VAR during 0.2–0.3 s, 600 VAR during 0.3–0.5 s, 0 VAR during 0.5–0.7 s, −600 VAR during 0.7–0.9 s, and 0 VAR during 0.9–1 s. Simulation results are shown in Figure 15 and Figure 16. Figure 15 shows the simulated waveforms of the grid inverter in case 1, in which the status of grid voltage is normal: Figure 15a shows the DC link voltage and reference; Figure 15b shows the active power; Figure 15c shows the reactive power and its reference; and Figure 15d shows the phase-a voltage and current waveforms. Figure 16 shows the simulated waveforms of the MG inverter in case 1: (a) active power and reference; (b) reactive power and reference; and (c) phase-a voltage and current waveforms.

#### 4.1.2. Case 2

**Figure 17:**
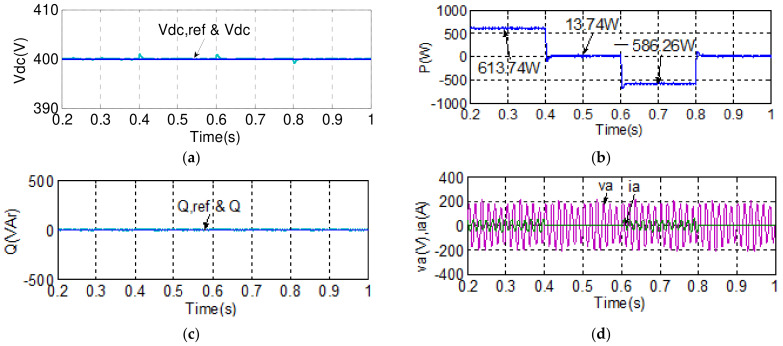
Simulated grid inverter waveforms in case 2: (**a**) DC link voltage and reference; (**b**) active power; (**c**) reactive power and reference; (**d**) phase-a voltage and current.

**Figure 18:**
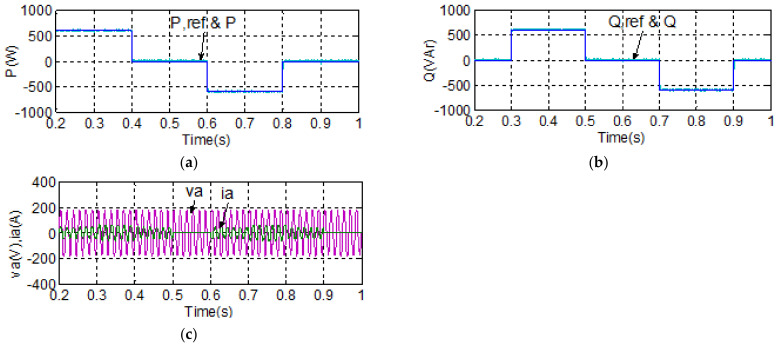
Simulated MG inverter waveforms in case 2: (**a**) active power and reference; (**b**) reactive power and reference; (**c**) phase-a voltage and current.

To demonstrate the advantage of using the proposed BTB inverter in achieving stable power flow control features in the MG inverter under a disturbed grid voltage, the simulation case 2 explores active/reactive power flow tracking control results of both grid and MG inverters under a voltage swell/sag scenario happening in the grid. The simulation scenario is described as follows: the voltage swell is set at 1.15 pu and the voltage sag is set at 0.85 pu. The voltage variation sequence is as follows: nominal voltage, then swell, then nominal, and then sag (1.2 periods each). In this case, the grid inverter maintains the DC link voltage at 400 V and reactive power at 0 VAR; active power command for the MG inverter is set at 600 W during 0.2–0.4 s, 0 W during 0.4–0.6 s, −600 W during 0.6–0.8 s, and 0 W during 0.8–1 s, while reactive power command for MG inverter is set at 0 VAR during 0.2–0.3 s, 600 VAR during 0.3–0.5 s, 0 VAR during 0.5–0.7 s, −600 VAR during 0.7–0.9 s, and 0 VAR during 0.9–1 s. Simulation results are shown in Figure 17 and Figure 18, where it can be clearly observed that both the controlled active and reactive powers of the MG inverter follow their individual references closely and arenot affected by the poor grid voltage condition. Figure 17 shows the simulated waveforms of the grid inverter in case 2, in which the grid voltage is disturbed: Figure 17a shows the DC link voltage and its reference; Figure 17b shows the active power; Figure 17c shows the reactive power and its reference; and Figure 17d shows the phase-a voltage and current waveforms. Figure 18 shows the simulated waveforms of the MG inverter in case 2: (a) active power and its reference; (b) reactive power and its reference; and (c) phase-a voltage and current waveforms.

### 4.2. Implementation Results

#### 4.2.1. Case 1

**Figure 19:**
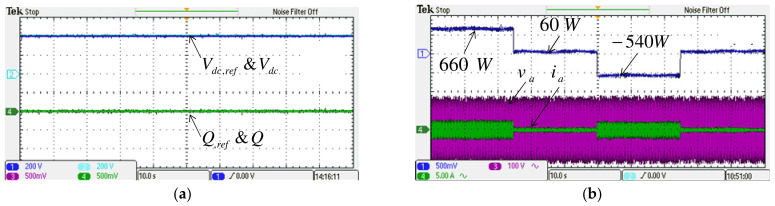
Implemented grid inverter waveforms in case 1: (**a**) DC link voltage and reference/reactive power and reference; (**b**) active power/a phase voltage and current.

**Figure 20:**
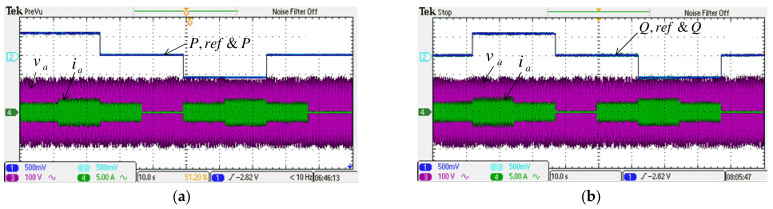
Implemented MG inverter waveforms in case 1 (**a**) active power and reference/reactive power and reference, (**b**) active power and reference/a phase voltage and current.

To verify the performance of the proposed control scheme, two typical experimental tests are carried out. The first test case aims to verify the results of simulation case 1. Results are shown in Figure 19 and Figure 20. Dynamic command tracking and the decoupling effect of the MG inverter active/reactive power control are the main focuses here. It can be observed in Figure 20a that power tracking is indeed very effectively performed.

#### 4.2.2. Case 2

**Figure 21:**
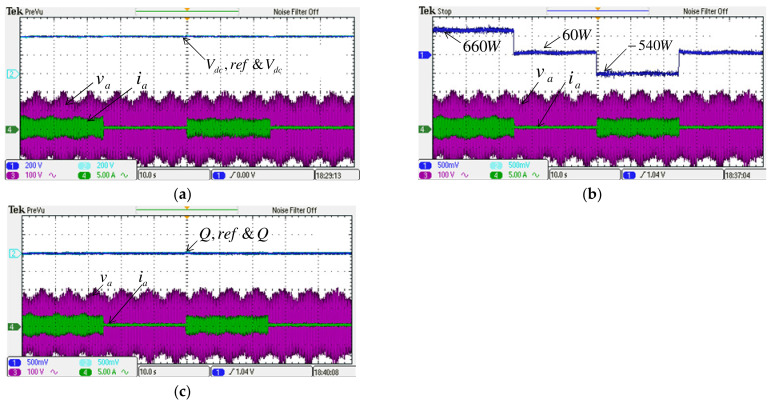
Implemented grid inverter waveforms in case 2: (**a**) DC link voltage and reference/a phase voltage and current; (**b**) active power/a phase voltage and current; (**c**) reactive power/phase-a voltage and current.

**Figure 22:**
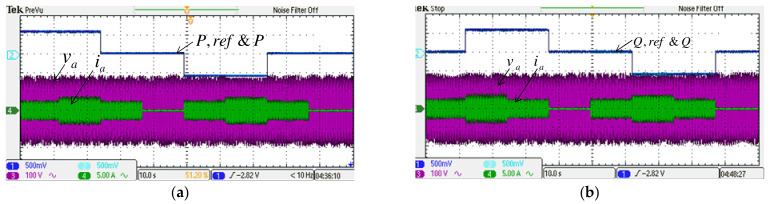
Implemented MG inverter waveforms in case 2: (**a**) active power and reference/a phase voltage and current; (**b**) reactive power and reference/phase-a voltage and current.

This case aims to verify the results of simulation case 2. Results are shown in Figure 21 and Figure 22. The MG’s PQ control performance during voltage sag/swell is the main focus here. It can be seen from Figure 22a,b that the PQ control performance of MG is successfully maintained under poor grid voltage conditions.

## 5. Conclusions

Usually, when an SS disconnects a MG from the grid to enable standalone operation, protection mechanisms are necessary to keep transient voltage from causing damage to the loads and the power converters in the MG. On the other hand, reconnecting the MG to the grid requires proper phase synchronization processes to ensure an acceptable transition. In this paper, the role of SS has been replaced by a BTB inverter system consisting of two SiC-based three-phase VSIs with identical architectures but different controllers and functions. It is worthwhile noting that that compared with an SS device, the proposed BTB configuration will induce the efficiency reduction problem; however, in some application cases, e.g., micro-grids in high-tech industry areas, preventing power quality issues, i.e., voltage dip, swell, imbalance, or harmonics are much more important than paying the price caused by efficiency reduction. In addition, the proposed system can also indirectly handle voltage and frequency differences between the grid and the MG, and the two systems do not impact each other when operating mode is being switched. As a result, the synchronization problem becomes easier to solve. The main advantage of the proposed system is that the grid and the MG can be isolated with the DC interface, so that the AC power flow control on one side does not directly affect the other, thereby improving overall PQ control performance and reliability. In the proposed MG control scheme, the main functions of grid inverter are to regulate DC link voltage and control reactive power, while the main function of MG inverter is to perform active/reactive power control. In practice, the proposed BTB inverter system can also perform various control functions for the MG. In terms of power flow management, the proposed BTB inverter can quickly and accurately adjust the required power level and direction, and the inverters on both sides are capable of performing independent reactive power compensation to improve the system’s PQ and thus reduce losses. In terms of eliminating possible adverse effects on the MG system, it has been proven that with the proposed control scheme, the stable operation of an MG can be maintained when the grid’s voltages are unstable due to abnormal situations. According to both simulation and implementation results, it can be verified that real-time mode-switching and stable power flow regulation for an MG can be achieved with the proposed control scheme.

## Figures and Tables

**Table 1:** System specifications.

Item	Value
Rated capacity, *S*	1 kVA
Grid lien voltage, *V_L-L_*	220 V
DC link voltage, *V_dc_*	400 V
Carrier amplitude, *V_tri_*	5 V
Grid frequency, *f_1_*	60 Hz
DC link capacitor, *C_dc_*	4080 μF
Filter inductor 1, *L_f1_*	3 mH
Filter inductor 2, *L_f2_*	0.5 mH
Grid inductance, *L_g1_*	1 mH
Filter capacitor, *C_f1_*	10 μF
Damping resistor, *R*	10 Ω
DC link voltage sensing factor, *K_dc_*	0.01 V/V
AC voltage sensing factor, *K_v_*	0.01 V/A

**Table 2:** Controller parameters of proposed grid inverter controllers.

Item	Value
Inner-loop current controller P gain	80
Inner-loop current controller $K_{pwm}^{-1}$ gain	0.025
DC link voltage controller P gain	3
DC link voltage controller I gain	1110
Reactive power controller P gain	1.6
Reactive power controller I gain	3040

**Table 3:** Controller parameters of proposed MG inverter controllers.

Item	Value
Inner-loop current controller P gain	80
Inner-loop current controller $K_{pwm}^{-1}$ gain	0.025
Active power controller P gain	1.6
Active power controller I gain	3040
Reactive power controller P gain	1.6
Reactive power controller I gain	3040

## Data Availability

No new data were created or analyzed in this study. Data sharing is not applicable to this article.
